# LIVING DONOR LIVER TRANSPLANT FOR COLORECTAL LIVER METASTASIS: THE FIRST CASE IN LATIN AMERICA

**DOI:** 10.1590/0102-672020180001e1468

**Published:** 2019-12-20

**Authors:** Eduardo de Souza M FERNANDES, Pal-Dag LINE, Felipe Pedreira de MELLO, Ronaldo Oliveira ANDRADE, Camila Liberato GIRÃO, Leandro Savattone PIMENTEL, Camilla CÉSAR, Tarik Soares SULEIMAN, Fabio Luís WAECHTER, Antonio Talvane T OLIVEIRA, Orlando Jorge M TORRES

**Affiliations:** 1 Department of Gastrointestinal Surgery - Rio de Janeiro Federal University and Liver Transplant Unit - São Lucas Hospital RJ, Brazil;; 2 Department of Oncology and Transplantation Medicine, Oslo University Hospital, Oslo, Norway;; 3 Department of Gastrointestinal Surgery, Santa Casa of Porto Alegre, RS, Brasil;; 4 Department of Gastrointestinal Surgery, Americas Hospital of Rio de Janeiro, RJ, Brazil;; 5 Department of Gastrointestinal Surgery-Hepatopancreatobiliary and Liver Transplant Unit, Maranhão Federal University, MA, Brazil.

**Keywords:** Liver metastases, Transplant for liver metastases, Living donor liver transplant, Liver transplant, Metástases hepáticas, Transplante em metástases hepáticas, Transplante de fígado de doador vivo, Transplante de fígado

## INTRODUCTION

Colorectal adenocarcinoma is a common malignancy around the world and synchronous or metachronous liver metastases will be observed in about 50% of these patients. Hepatic resection is a potentially curative treatment for metastases from colorectal cancer^1^ ^,^ ^2^. However, only about 20% of the patients are suitable for resection, and recurrence occur in the majority of these patients and they are candidates for palliative chemotherapy. Liver transplant has been performed for liver tumors in well selected patients, mainly hepatocellular carcinoma, liver metastases from neuroendocrine tumors and peri-hilar cholangiocarcinoma emerging the concept of transplant oncology. Complete surgical resection is the treatment of choice for patients with liver metastases, but in a large proportion it is not possible to obtain a complete R0 resection. In 2006 the Oslo group started the first trial on liver transplant for patients with colorectal liver metastases (SECA I study). The inclusion criteria were R0 primary colorectal resection, unresectable liver metastases, no extrahepatic disease, at least six weeks of chemotherapy and an Eastern Cooperative Oncology Group (ECOG) performance status 0-1^2^ ^,^ ^3^. Twenty-one patients with unresectable colorectal liver metastases (u-CRLM) were included. The overall survival rate at five years was 60% with a median survival time of 27 months. Notwithstanding the disease free survival rate was 35% at one year and all patients got relapse if observed up to three years, mainly in the form of lung metastases which were slow growing and most often resectable. Some factors were identified as related to worse prognosis (the Oslo Criteria) and include: 1) time from primary cancer surgery <2 years; 2) progressive disease on chemotherapy; 3) maximum tumor diameter >5.5 cm; and 4) CEA levels >80 μg/l. Beside Norway, liver transplant for colorectal liver metastasis have been performed in Japan, France, Canada, Portugal, Turkey, and Germany^2^ ^,^ ^4^ ^,^ ^5^. Very recently the Oslo group reported the preliminary results of SECA II trial, indicating that a five year overall survival of about 80% may be obtained if stricter selection criteria for liver transplant in this patient cohort are used^6^. Nowadays, the majority of liver transplant reported for u-CRLM utilize deceased donor liver transplant (DDLT). In Brazil DDLT is not possible due to organ shortage problem and living donor liver transplant (LDLT) seems to be the only available alternative. 

The aim of this study was to present the first case of patient with colorectal liver metastases underwent LDLT in Latin America. 

## CASE REPORT

### Recipient

The recipient was a 68 year-old male, height 1.78 m, weight 96 kg, BMI 30.3 with synchronous unresectable CRLM from colorectal cancer located in the left colon. After neoadjuvant chemotherapy with FOLFOX, he underwent robotic colectomy and resection of multiple bilateral liver metastases in November 2017. Adjuvant chemotherapy included FOLFOX and FOLFIRI. In August 2018 the liver metastases recurred and he underwent left hepatectomy including middle hepatic vein, intraoperative ultrasound and radiofrequency ablation of one lesion in segment 6 followed by chemotherapy with FOLFIRI. Another liver recurrence occurred in December 2018. A Kras wild type was identified and a new chemotherapy regimen with FOLFIRI and Cetuximab was started. A PET-CT confirmed liver-only metastatic disease. The CEA at that time was 8.3 and CA 19-9 14. The calculated Standard Liver Volume (SLV) according to the formula 11.5 x weight + 334 was 1,438 cc^3^ and the corresponding 40-50% of SLV was 575-719 cc^3^. During the tumor board liver transplant was suggested. All the steps of the procedure were discussed with the patient and family including risks and success rate. 

### Donor

The donor was the son of the recipient, a 41 year old man, height 1,86; weight 78 kg, BMI 23. Right lobe was used without middle hepatic vein and the volume was 935 cc^3^ (841g). GRWR was 0.841/96=0.87. Residual left lobe (RLL) was 35.5%.

### Technical aspects

**FIGURE 1:**
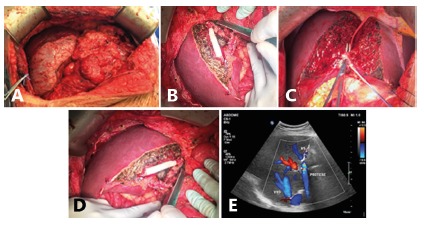
A) Metastatic liver of the recipient; B) splitting the liver of the donor, preserving the middle hepatic vein with the donor; C) venoplasty with 10 mm PTFE prosthesis; D) right liver is implanted in the recipient; E) Doppler after liver transplant (VHD=right hepatic vein and protese=prosthesis).

Explorative laparotomy was performed in the recipient and no extrahepatic disease was observed in the abdomen (Figure 1A). The liver pedicle was dissected and the portal vein, hepatic artery and bile duct were isolated. The vena cava was dissected and isolated the right hepatic vein, preparing for total hepatectomy. 

In the donor the strategy was defined as right hepatectomy without middle hepatic vein and preserving V5 and V8 to perform anastomosis with 10 mm PTFE prosthesis during the back-table and venoplasty with the right hepatic vein (Figure 1B and 1C). The modified right lobe was implanted under partial clamping of the inferior vena cava and reconstruction of the Makuuchi vein (Figure 1D and 1E).

Postoperative course was uneventful for both recipient and donor. The donor stayed at the intensive care unit for one day and the recipient was discharged from the intensive care unit after two days. The total length of hospital stay for both recipient and donor was six days.

## DISCUSSION

Palliative chemotherapy is the only effective treatment for patients with unresectable liver metastases. Recently many efforts have been made to increase resectability, including portal vein embolization, two stage hepatectomy and ALPPS. In the design of the SECA studies from Oslo University Hospital they initially used strict inclusion criteria to select candidates for liver transplantation. Due to low number of referrals in the first trial they decided to simplify the inclusion criteria and more patients were considered for inclusion. This led to great heterogeneity related to tumor load, biology and chemotherapy in the SECA 1 study^5^ ^,^ ^7^ ^,^ ^11^. Nevertheless, the outcomes were far better than expected. In the SECA 2 trial transplantation was limited to extreme selected candidates with low perceived oncological risk, and the estimated five year survival at five years was 83%, comparable to standard indications for liver transplantation^2^ ^,^ ^5^ ^,^ ^7^ ^,^ ^11^. 

On this background several centers are currently considering liver transplantation as a possible option for patients with u-CRLM; limited survival benefit and organ shortage limits the applicability and implementation for this procedure in most countries. However, in living donor liver transplant we do not use the liver from the patient in waiting list, minimizing the problem of organ shortage. In the present study the donor was his son, and he was prepared for the procedure and in good clinical conditions. To try to ensure maximal transplant benefit the patient was included according to the Oslo criteria for liver transplant of CRLM.^1^ ^,^ ^2^ ^,^ ^4^ ^,^ ^7^.

Thanks to a better knowledge of the biology of metastatic disease, proper selection of patients with imaging techniques, effective chemotherapy and improved immunosuppressive agents, improved outcomes following liver transplantation for malignant disease may be obtained. Nowadays, liver transplant for liver metastases has been performed in some countries besides Norway^1^ ^,^ ^2^ ^,^ ^4^. The transplanted patient maintain a good quality of life for a long period of time and negative impact of the immunosuppression agents has not been observed. In this study the patient was clinically asymptomatic after 60 days of the liver transplant^2,3,8,12.^. 

For selected patients with nonresectable liver metastases without extrahepatic disease, liver transplant may increase overall survival when compared with chemotherapy according to two prospective studies (SECA study and NORDIC VII study) with many similarities and some differences between groups. A randomized multicentric trial is ongoing to evaluate more precisely the role of liver transplant for unresectable colorectal liver metastases^1^ ^,^ ^3^ ^-^ ^6^. 

Liver transplant for patients with hepatocellular carcinoma included in the Milan criteria has been well established as the primary treatment and the 5-year overall survival rate is 70-80 per cent^1,2,4,11,12^ . In selected (low-risk group) patients with colorectal cancer, the survival after five years is similar or even better than patients with hepatocellular carcinoma. However, some of the patients with hepatocellular carcinoma die from causes other than recurrent disease, different from patients with colorectal metastases. It is necessary to identify patients with probability of long overall survival after liver transplantation based on clinical criteria. Dueland et al.^2^ ^,^ ^3^ observed that four patients with metachronous disease and negative node in the primary tumor are still alive after 6-10 years of the transplant suggesting that by refining the selection of patient even better long term survival and possible cure in a proportion of cases may be achieved^1^ ^,^ ^2^ ^,^ ^3^ ^,^ ^8^ ^,^ ^12^.
